# Chromosome assembly of *Collichthys lucidus*, a fish of Sciaenidae with a multiple sex chromosome system

**DOI:** 10.1038/s41597-019-0139-x

**Published:** 2019-07-24

**Authors:** Mingyi Cai, Yu Zou, Shijun Xiao, Wanbo Li, Zhaofang Han, Fang Han, Junzhu Xiao, Fujiang Liu, Zhiyong Wang

**Affiliations:** 1 Key Laboratory of Healthy Mariculture for the East China Sea, Ministry of Agriculture and Rural Affairs; Fisheries College Jimei University, Xiamen, Fujian China; 2 Laboratory for Marine Fisheries Science and Food Production Processes, Qingdao National Laboratory for Marine Science and Technology, Qingdao, China; 3 School of Computer Science and Technology, Wuhan University of Technology, Wuhan, Hubei China; 4 Wuhan Frasergen Bioinformatics, East Lake High-Tech Zone, Wuhan, China

**Keywords:** Molecular evolution, Genome

## Abstract

*Collichthys lucidus* (*C*. *lucidus*) is a commercially important marine fish species distributed in coastal regions of East Asia with the X_1_X_1_X_2_X_2_/X_1_X_2_Y multiple sex chromosome system. The karyotype for female *C*. *lucidus* is 2n = 48, while 2n = 47 for male ones. Therefore, *C*. *lucidus* is also an excellent model to investigate teleost sex-determination and sex chromosome evolution. We reported the first chromosome genome assembly of *C*. *lucidus* using Illumina short-read, PacBio long-read sequencing and Hi-C technology. An 877 Mb genome was obtained with a contig and scaffold N50 of 1.1 Mb and 35.9 Mb, respectively. More than 97% BUSCOs genes were identified in the *C*. *lucidus* genome and 28,602 genes were annotated. We identified potential sex-determination genes along chromosomes and found that the chromosome 1 might be involved in the formation of Y specific metacentric chromosome. The first *C*. *lucidus* chromosome-level reference genome lays a solid foundation for the following population genetics study, functional gene mapping of important economic traits, sex-determination and sex chromosome evolution studies for Sciaenidae and teleosts.

## Abstract (summary)

**Table:** 

Design Type(s)	sequence assembly objective • sequence annotation objective • transcription profiling design
Measurement Type(s)	whole genome sequencing assay • transcript expression assay
Technology Type(s)	DNA sequencing • RNA sequencing
Factor Type(s)	organism part
Sample Characteristic(s)	Collichthys lucidus

Machine-accessible metadata file describing the reported data (ISA-Tab format)

## Background & Summary

*Collichthys lucidus* (*C*. *lucidus*, FishBase ID: 23635, NCBI Taxonomy ID: 240159, Fig. 1), also called spiny head croaker or big head croaker, belongs to Perciformes, Sciaenidae, *Collichthys* and is mainly distributed in the shore waters of the northwestern Pacific, covering from the South China Sea to Sea of Japan^1^. *C*. *lucidus* is a commercially important marine fish species with high market value and has been widely consumed in coastal regions in China^2^.

**Fig. 1:**
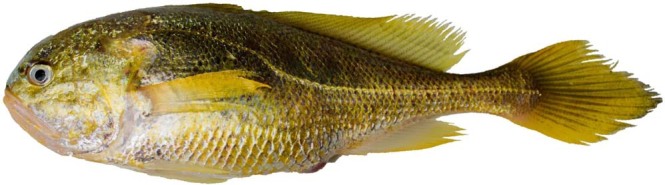
A picture of *Collichthys lucidus* used for the genome sequencing.

At present, the research on *C*. *lucidus* mostly focused on phylogeny and population genetics^3–7^. *C*. *lucidus* exhibits apparent sex dimorphism on the growth rate that the female grow much faster than male ones; therefore, the understanding of its sex-determination would facilitate the development of the sex control technique in aquaculture industry to increase the annual yield. More interesting, our previous cytogenetic study showed that female *C*. *lucidus* had 24 pairs of acrocentric chromosomes (2n = 48a, NF = 48), while male ones had 22 pairs of acrocentric chromosomes, two monosomic acrocentric chromosomes and one metacentric chromosome (2n = 1 m + 46a, NF = 48)^8^. There is an X_1_X_1_X_2_X_2_/X_1_X_2_Y mechanism of the sex-chromosome type in *C*. *lucidus*, while Y is a unique metacentric chromosome in the male karyotype. Although multiple sex chromosome systems are found in several Perciformes species^9^, *C*. *lucidus* is the first reported case in the Sciaenidae species. At present, researches on the sex determination and differentiation mechanism in the Sciaenidae species are still lacking. Previous studies showed that no heterotropic chromosome was found in large yellow croaker (*Larimichthys crocea*) and spotted maigre (*Nibea albiflora*)^10,11^. As a close-related species in the same family, the chromosome comparison might provide insights into chromosome evolution among the species and the relationship to the evolution of sex-determination in Sciaenidae.

To obtain high-quality chromosome sequences of *C*. *lucidus*, we applied a combined strategy of Illumina, PacBio and Hi-C technology^12^ to sequence the genome of *C*. *lucidus* and reported the first chromosome-level assembly of this important species. The genome will be used for the functional gene mapping of the economic traits and the sex-determination of *C*. *lucidus*, as well as in the chromosome evolution investigations among Sciaenidae and teleosts.

## Methods

### Sample collection

A female wild-caught adult *C*. *lucidus* in Baima Harbor, Ningde, Fujian, China (26.7328°N, 119.7329°E) was used for the genome sequencing and assembly. The reason we chose a female sample is that the heterotropic chromosome in male might increase the technical challenge of genome assembly, especially for X_1_ and X_2_ chromosomes. Muscle, eye, brain, heart, liver, spleen, kidney, head kidney, gonad, stomach and intestines of the fish were harvested. All samples were rinsed with 1×PBS (Phosphate Buffered Solution) solution quickly, frozen with liquid nitrogen over 24 hours and then stored in −80 °C before sample preparation.

### DNA extraction and sequencing

Phenol/chloroform extraction method was used in DNA molecules extraction from muscle tissues. The DNA molecules were used for sequencing on the Illumina (Illumina Inc., San Diego, CA, USA) and PacBio sequencing platform (Pacific Biosciences of California, Menlo Park, CA, USA). DNA library construction and sequencing in the Illumina sequencing platform were carried out according to the manufacturer’s instruction as in the previous study^13^. Briefly, the DNA extracted from muscle samples were randomly sheared to 300–350 bp fragments using an ultrasonic processor and paired-end library was constructed through the steps of end repair, poly(A) addition, barcode index, purification, and PCR amplification. The constructed DNA library was sequenced by Illumina HiSeq X platform in 150 PE mode. As a result of Illumina sequencing, we obtained 52.0 Gb raw genome data for *C*. *lucidus*. After the quality filtering, 51.35 Gb clean reads were retained as summarized in Table 1. Meanwhile, Genomic DNA molecules of *C*. *lucidus* were also used for one 20 kb library construction. Eleven flow cells were used in the PacBio Sequel platform to generate 90.7 Gb (109.3× coverage) polymerase sequencing data. After filtering adaptors in the sequencing reads, 90.5 Gb long reads were obtained for the following genome assembly (Table 1).

**Table 1:** Sequencing data used for the *C*. *lucidus* genome assembly.

Types	Method	Library size (bp)	Clean data (Gb)	length (bp)	coverage (×)
Genome	Illumina	300–350	52.0	150	62.6
Genome	Pacbio	20,000	90.5	14,002	109.0
Genome	Hi-C	—	193.1	150	232.7
Transcriptome	Illumina	250–300	9.8	150	—

The coverage was calculated using an estimated genome size of 830 Mb.

### RNA extraction and sequencing

Transcriptome of *C*. *lucidus* was also sequenced in this work for the gene prediction after the genome assembly. Muscle, eye, brain, heart, liver, spleen, kidney, head kidney, gonad, stomach and intestines tissues collected before from the same individual were used for RNA extraction with TRIZOL Reagent (Invitrogen, USA). The RNA molecules extracted from tissues were then equally mixed for RNA sequencing. According to the protocol suggested by the manufacturer, RNA sequencing library was constructed as the previous study^14^ and sequenced by Illumina HiSeq X Ten in 150PE mode (Illumina Inc., San Diego, CA, USA). Finally, ~9.8 Gb RNA-seq data were obtained (Table 1).

### Genome survey and contig assembly

**Fig. 2:**
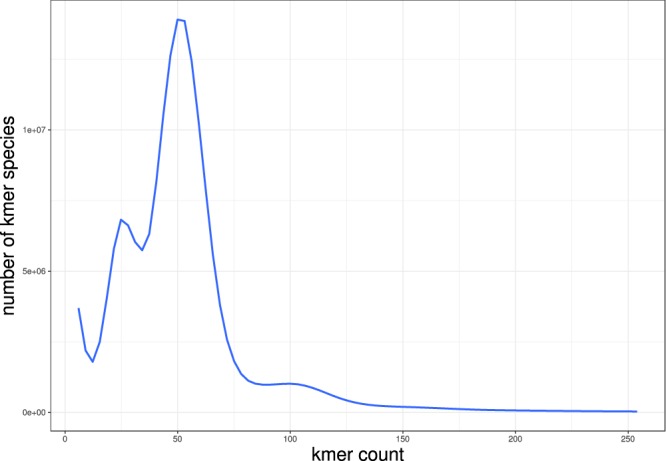
*K*mer frequency of *C*. *lucidus*. Note that the first, second and third peak was composed of the homozygous, heterozygous and repeated *K*mers, respectively.

The genome size of the genome of *C*. *lucidus* was estimated with Illumina sequencing data using *K*mer-based method implemented in GCE (v1.0.0)^15^ before genome assembly. Using *K*mer size of 17, we obtain a *K*mer frequency distribution for *C*. *lucidus* (Fig. 2). The genome size was estimated using the following equation: *G* = (*L* − *K* + 1) × *n*_*base*_/(*C*_*Kmer*_ × *L*), Where *G* is the estimated genome size, *n*_*base*_ is the total count of bases, *C*_*Kmer*_ is the expectation of *K*mer depth, *L* and *K* is the read length and *K*mer size. Since *K*mers with the depth smaller than three were likely from sequencing errors, we, therefore, revise the genome size by the following method: *G*_*revise*_ = *G* × (1 – *Error Rate*). As a result, we estimated female *C*. *lucidus* genome size of 830 Mb with the heterozygosity of 0.81% and the whole-genome average GC content of 42%.

To assembly contig sequences using long-read data, the software Falcon v0.30^16^ was used for the contig assembling of the female genome of *C*. *lucidus* with default parameters. The genome assembly was performed by following steps in Falcon: First, daligner^17^ was used to generate read alignments, and the consensus reads were generated. Then, the overlap information among error-corrected reads were generated by daligner. Finally, a directed string graph was constructed from overlap data, and contig path were resolved by the string graph. Two round of sequence polishing was performed as follows: the assembled genome sequence was first polished with arrow^18^ using PacBio long reads, and Pilon^19^ was then used with Illumina sequencing data. In the end, we yielded a final genome contig assembly of *C*. *lucidus* with a total length 877.4 Mb with 2,912 contigs and a contig N50 of 1.10 Mb. (Table 2).

**Table 2:** Assembly statistics of *C*. *lucidus*.

Sample ID	Contig Length (bp)	Contig number
Total	877,428,965	2,912
Max	9,855,977	—
Number >=2000bp	—	2,853
N50	1,098,566	210
N60	794,488	305
N70	545,261	437
N80	319,460	646
N90	152,174	1,044

### Chromosome assembly using Hi-C data

To obtain a chromosome assembly of *C*. *lucidus*, we applied the Hi-C technique to generate the interaction information among contigs. 1 g muscle tissue was used for Hi-C library construction. The processes of crosslinking, lysis, chromatin digestion, biotin marking, proximity ligations, crosslinking reversal, and DNA purification steps were used in previous studies^20^. The Hi-C library was sequenced in Illumina HiSeq X Ten platform, and 193.1 Gb Hi-C reads were generated (Table 1). The reads were aligned to the assembled contig sequences using Bowtie software, and the alignment was filtered as our previous study^21^. The interaction matrix among contig was generated, and Lachesis^22^ was then applied to anchor contigs into chromosomes with the agglomerative hierarchical clustering method. Finally, we successfully scaffolded 2,134 contigs into 24 chromosomes, representing 96.86% of the total assembled genome. The contig and scaffold N50 of the chromosome assembly was 1.1and 35.9 Mb, respectively. We noted that there are 865 contigs cannot reliably be anchored to any chromosome, and the N50 length of unanchored contigs was 49.4 kb, which was significantly smaller than that of 1.16 Mb for anchored contigs.

### Gene prediction and functional annotation

The repetitive sequences in the *C*. *lucidus* genome sequences were annotated through a combination of homology prediction and *ab initio* prediction. RepeatMasker (http://www.repeatmasker.org/)^23^ and RepeatProteinMask were applied for searching against RepBase database (http://www.girinst.org/repbase). We used Tandem Repeats Finder (TRF)^24^ and LTR-FINDER^25^ with default parameters for *ab initio* prediction. As a result, we identified 304.40 Mb of the assembled *C*. *lucidus* genome as repetitive elements, accounting for 34.68% of the total genome sequences. The repetitive elements were masked in the *C*. *lucidus* genome sequences, and the repeat-masked genome was used for the gene prediction.

The protein-coding gene annotation was identified by a combined strategy of homology-based prediction, *ab initio* prediction, and transcriptome-based prediction method. The protein sequences of several teleosts, including *Danio rerio* (GCF_000002035.6), *Dicentrarchus labrax* (GCA_000689215.1), *Gasterosteus aculeatus* (GCA_000180675.1), *Oryzias latipes* (GCF_002234675.1) and *Takifugu rubripes* (GCF_000180615.1) were mapped upon the assembled *C*. *lucidus* genome using TBLASTN^26^. The alignments were conjoined by Solar software^27^. GeneWise^28^ was used to predict the exact gene structure of the corresponding genomic region on each BLAST hit. Furthermore, the sequences from RNA-seq were aligned to the assembled *C*. *lucidus* genome to identify potential exon regions by TopHat^29^ and Cufflinks^30^. Then, Augustus^31^ was also used to predict coding regions in the repeat-masked genome sequences. All these results were merged by MAKER^32^, leading to a total 28,602 protein-coding genes (Table 3). After homolog searching against to NCBI non-redundant protein (NR)^33^, TrEMBL^34^, Gene Ontology (GO)^35^, SwissProt^34^, Kyoto Encyclopedia of Genes and Genomes (KEGG)^36^, InterPro^37^, 28,032 (98.01%) protein-coding genes were annotated with at least one public functional database (Table 4).

**Table 3:** General statistics of predicted protein-coding genes.

Gene set		Number	Average transcript length (bp)	Average CDS length (bp)	Average exons per gene	Average exon length (bp)	Average intron length (bp)
***De novo***	**Augustus**	32,502	11,378.88	1,494.29	8.52	175.44	1,314.88
	**Genscan**	40,805	15.596.28	1,560.39	8.56	182.21	1,855.72
**Homolog**	***D.*** ***rerio***	52,244	9,049.21	1,076.27	5.56	193.69	1,749.76
	***D.*** ***labrax***	48,861	7,508.49	1,028.16	5.79	177.46	1,351.80
	***G.*** ***aculeatu***	45,957	7,811.18	1,035.02	6.04	171.27	1,447.46
	***O.*** ***latipes***	44,650	8,137.02	1,036.88	5.91	175.59	1,405.38
	***T.*** ***rubripes***	43,159	8,366.10	1,046.02	6.21	168.48	1,401.06
**trans.orf/RNAseq**		**18,058**	11,694.21	1,095.81	7.62	317.99	1,401.06
**MAKER**		**28,602**	13,241.72	1,673.58	9.74	207.05	1,284.21

**Table 4:** General statistics of gene function annotation.

Type		Number	Percent(%)
**Total**		28,602	100
**Annotated**	**InterPro**	24,918	87.12
	**GO**	18,942	66.23
	**KEGG**	17,806	62.25
	**Swissprot**	26,038	91.04
	**TrEMBL**	27,883	97.49
	**NR**	27,996	97.88
**Annotated**		28,032	98.01
**Unannotated**		570	1.99

### Repeat distribution and potential sex-determination gene identification

**Fig. 3:**
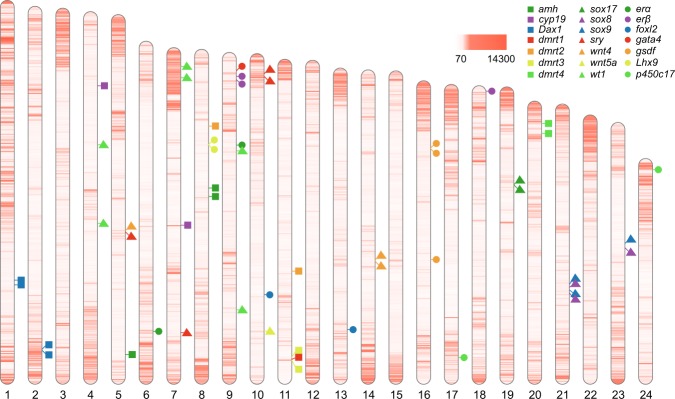
Repetitive element distribution and potential sex-determination gene identification in the chromosomes of *C*. *lucidus*. The color bar represented the density of repetitive elements (number per 100 kb) along the genome and 21 key genes involving in teleost sex-determination that reported in previous studies were identified and label on chromosomes.

The distribution of repetitive elements along chromosomes was plot in Fig. 3. The repeats were generally concentrated at the two ends of the chromosomes, especially on the beginning end of the chromosome 1 in the assembled *C*. *lucidus* genome. Our previous cytogenetic analysis revealed that a chromosome with ending massive repeats was involved in the formation of Y specific metacentric chromosome^8^, we therefore speculated that chromosome 1 might be one of the two chromosomes in the sex chromosome fusion. Twenty one potential key genes in sex development of teleost were identified along the assembled *C*. *lucidus* genome (Fig. 3), facilitating the gene expression and functional studies aiming to the deciphering the sex-determination of *C*. *lucidus*. We identified the only one copy of *Dmrt1* gene (dsx- and mab-3 related transcription factor 1) in the chromosome 11. Our previous studies on the studies of *L*. *crocea*^10^ and *N*. *albiflora*^11^ revealed that *Dmrt1* was a key gene in sex-determination of two species, we therefore speculated the *Dmrt1* gene might also play an central role in sex-determination process of *C*. *lucidus*. The sequences of chromosomes and genes provided valuable resource for the following sex-determination investigations.

## Data Records

The genomic Illumina sequencing data were deposited in the Sequence Read Archive at NCBI SRR8208332^38^.

The genomic PacBio sequencing data were deposited in the Sequence Read Archive at NCBI SRR8142901^39^.

The transcriptome Illumina sequencing data were deposited in the Sequence Read Archive at NCBI SRR8208331^40^.

The Hi-C sequencing data were were deposited in the Sequence Read Archive at NCBI SRR8208301^41^.

The final chromosome assembly were deposited in the GenBank at NCBI SCMI00000000^42^.

The genome annotation file is available within figshare^43^.

The sequences of potential sex-determination genes identified from the assembled *C.* *lucidus* genome is available within figshare^44^.

## Technical Validation

The quality of the DNA molecules was checked by agarose gel electrophoresis, showing the main band around 20 kb, and the extracted DNA spectrophotometer ratios (SP) were 260/280 ≥ 1.8.

The quality of the purified RNA molecules were checked by Nanodrop ND-1000 spectrophotometer (LabTech, USA) as the absorbance >1.7 at 260 nm/280 nm and 2100 Bioanalyzer (Agilent Technologies, USA) as the RIN of 8.0.

The raw reads from Illumina sequencing platform were cleaned using FastQC^45^ and HTQC^46^ by the following steps: (a) filtered reads with adapter sequence; (b) filter PE reads with one reads more than 10% N bases; (c) filtered PE reads with any end has more than 50% inferior quality (< = 5) bases.

**Fig. 4:**
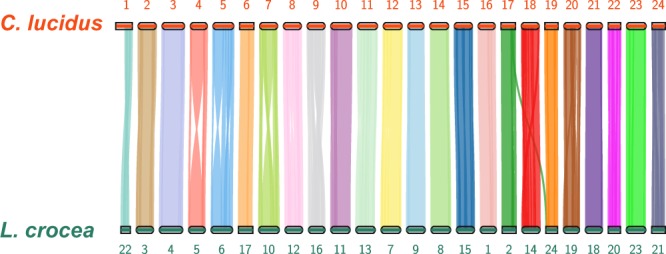
Chromosome comparison of *C*. *lucidus* to *L*. *corcea *using protein-coding genes synteny. The chromosome id of *C*. *lucidus* were sorted by the sequence lengths.

The quality of the assembled genome were validated on terms of the completeness, accuracy and conservation synteny. Firstly, the completeness of the genome sequences was validated by the alignments of PacBio long reads.Minimap2^47^ with default parameters was applied to map the CLR (Continuous Long Reads) subreads of *C*. *lucidus* back to the final chromosome assembly. We found that about 96.2% of the long reads could be aligned to the assembled genome, and the average depth of the alignment along the genome was 103 × . More than 99.78% and 98.1% of the genome sequences were aligned by at least 1× and 20× coverage, respectively. Secondly, we further confirmed the completeness of the assembled genome using BUSCO v3.0^48^. As a result, 97.6% and 97.4% BUSCO genes were completely or partially identified in the assembled *C*. *lucidus* genome with the vertebrate and actinopterygii database, respectively. Thirdly, the accuracy of the genome assembly was evaluated by variants calling using Illumina data. The short reads were mapped to the genome sequences with BWA^49^. The insertion length distribution with one peak agreed well with our experimental design, suggesting the accuracy of the genome assembly. SNP calling with read alignments in GATK^50^ resulted in 2,593,807 heterozygous and 11,282 homozygous SNP loci along the genome sequences, suggesting the base-level accuracy of 99.999% for the genome assembly. Fourthly, the conservation synteny between *C*. *lucidus* and *L*. *crocea*^51^ were compared to validate the chromosome assembly. We observed a highly conserved synteny and strict correspondence of chromosome assignment (Fig. 4).

## ISA-Tab metadata file

Download metadata file

## Data Availability

No specific code were developed in this work. The data analysis were performed according to the manuals and protocols provided by the developer of the corresponding bioinformatics tools.
